# The Challenge of Measuring Sweet Taste in Food Ingredients and Products for Regulatory Compliance: A Scientific Opinion

**DOI:** 10.1093/jaoacint/qsac005

**Published:** 2022-01-18

**Authors:** Dustin E Starkey, Zhuzhu Wang, Kommer Brunt, Lise Dreyfuss, Philip A Haselberger, Stephen E Holroyd, Kaushik Janakiraman, Prabhakar Kasturi, Erik J M Konings, David Labbe, Marie E Latulippe, Xavier Lavigne, Barry V McCleary, Salvatore Parisi, Tony Shao, Darryl Sullivan, Marina Torres, Sudhakar Yadlapalli, Ioannis Vrasidas

**Affiliations:** 1 Abbott Nutrition, 3300 Stelzer Rd, Columbus, OH 43219, USA; 2 Abbott Nutrition, 1800 South Oak St, Suite 210 Champaign, IL61820, USA; 3 University of Illinois, Department of Food Science and Human Nutrition, 1302 W. Pennsylvania Ave, Urbana, IL 61801, USA; 4 Rotating Disc b.v, Spoorlaan 31, 9753HVHaren, The Netherlands; 5 SAM Sensory and Marketing International, 46 rue Armand Carrel, 75019 Paris, France; 6 Fonterra Research and Development Centre, Private Bag 11029, Palmerston North4 442, New Zealand; 7 Reckitt Health, 60 Radarweg, 1043NT Amsterdam, The Netherlands; 8 PepsiCo R&D, 617, W. Main St, Barrington, IL 60010, USA; 9 Société des Produits Nestlé SA Nestlé Institute of Food Safety and Analytical Sciences, EPFL Innovation Park, Bâtimon G, 1015 Lausanne, Switzerland; 10 Société des Produits Nestlé SA Nestlé Institute of Material Sciences, Rte du Jorat 57, 1000 Lausanne 26, Switzerland; 11 Institute for the Advancement of Food and Nutrition Sciences, 740 15th St NW, #600, Washington DC 20005, USA; 12 Abbott Nutrition, Park Lane, Culliganlaan 2B, 1831 Diegem, Belgium; 13 Eden Rd, Greystones, Murrumburrah, County Wicklow A63YW01, Ireland; 14 Lourdes Matha Institute of Hotel Management and Catering Technology, Kuttichal PO, Thiruvananthapuram, Kerala 695574 India; 15 Eurofins Scientific, N2743 Butternut Rd, Pyonette, WI 53955, USA; 16 Departamento de Desarrollo de Métodos Analiticos, Laboratorio Tecnológico del Uruguay LATU, Avenida Italia, 6201 11500 Montevideo, Uruguay; 17 FirstSource Laboratory Solutions LLP (Analytical Services), First Floor, Plot No- A1/B, IDA Nacharam Cross Rd., Hyderabad 500076 India; 18 Purperhoedenveem 94, 1019HM Amsterdam, The Netherlands

## Abstract

The Codex Alimentarius Commission, a central part of the joint Food and Agricultural Organization/World Health Organizations Food Standards Program, adopts internationally recognized standards, guidelines, and code of practices that help ensure safety, quality, and fairness of food trade globally. Although Codex standards are not regulations per se, regulatory authorities around the world may benchmark against these standards or introduce them into regulations within their countries. Recently, the Codex Committee on Nutrition and Foods for Special Dietary Uses (CCNFSDU) initiated a draft revision to the Codex standard for follow-up formula (FUF), a drink/product (with added nutrients) for young children, to include requirements for limiting or measuring the amount of sweet taste contributed by carbohydrates in a product. Stakeholders from multiple food and beverage manufacturers expressed concern about the subjectivity of sweetness and challenges with objective measurement for verifying regulatory compliance. It is a requirement that Codex standards include a reference to a suitable method of analysis for verifying compliance with the standard. In response, AOAC INTERNATIONAL formed the Ad Hoc Expert Panel on Sweetness in November 2020 to review human perception of sweet taste, assess the landscape of internationally recognized analytical and sensory methods for measuring sweet taste in food ingredients and products, deliver recommendations to Codex regarding verification of sweet taste requirements for FUF, and develop a scientific opinion on measuring sweet taste in food and beverage products beyond FUF. Findings showed an abundance of official analytical methods for determining quantities of carbohydrates and other sweet-tasting molecules in food products and beverages, but no analytical methods capable of determining sweet taste. Furthermore, sweet taste can be determined by standard sensory analysis methods. However, it is impossible to define a sensory intensity reference value for sweetness, making them unfit to verify regulatory compliance for the purpose of international food trade. Based on these findings and recommendations, the Codex Committee on Methods of Analysis and Sampling agreed during its 41st session in May 2021 to inform CCNFSDU that there are no known validated methods to measure sweetness of carbohydrate sources; therefore, no way to determine compliance for such a requirement for FUF.

The Codex Standard (CXS) for follow-up formula (FUF; CXS 156–1987) has been under discussion for revision at the Codex Committee on Nutrition and Foods for Special Dietary Uses (CCNFSDU) since 2013. CCNFSDU has made significant progress and extensive modifications according to the latest science, and the entire composition is nearly complete. The draft revised standard is now divided into two parts for products covering the age ranges of 6–12 and 12–36 months.

The standard will become one of the strictest commodity standards of Codex Alimentarius in terms of carbohydrate composition. It will provide ranges for all macronutrients, including carbohydrates and total energy content, and further specify: *(1)* that lactose is the preferred source of carbohydrate; *(2)* a strict maximum limit of mono- and disaccharides excluding lactose at 2.5 g/100 kcal; *(3)* that sucrose and/or fructose should not be added; and *(4)* that the additive categories of sweeteners and flavor enhancers are prohibited. These requirements are measurable and justifiable for verifying compliance with the standard provisions. However, during the last two years, CCNFSDU has also explored the possibility of measuring or further restricting sweetness. The concept of sweetness is mentioned in three parts of Section B of the FUF standard (CXS 156–1987), which specifically addresses drink/product for young children with added nutrients/drink for young children aged 12–36 months. Two references in B.3.1 (footnote 5) specify for products based on non-milk protein that includes *(1)* carbohydrate sources that have no contribution to sweet taste should be preferred and *(2)* in no case be sweeter than lactose. Lastly, one reference in B.3.2.4 (optional ingredients) specifies *(3)* ingredients shall not be added with the purpose of imparting or enhancing a sweet taste.

In 2019, the AOAC INTERNATIONAL delegation intervened during CCNFSDU41 to express doubt about the ability to analytically measure and enforce a requirement for sweet taste objectively. It is indeed a requirement for Codex standards to include a reference to a suitable method of analysis for verifying compliance with the standard. CCNFSDU agreed to ask the Codex Committee on Methods of Analysis and Sampling (CCMAS) for guidance by referring a question to CCMAS41 on whether there are internationally validated methods to measure the sweetness of carbohydrate sources. In response, AOAC launched an Ad Hoc Expert Panel on Sweetness in November 2020 at the request of stakeholders who expressed concern about the inability to verify compliance with sweetness requirements in the revised CXS. The panel was composed of experts from infant formula and food industries, academia, International Organization for Standardization (ISO) and International Dairy Federation (IDF). They assessed the landscape of international analytical and sensory analysis methods for determining sweet taste of carbohydrate sources, with an objective to develop comments for CCMAS. The panel summarized its findings and recommendations in a Codex Conference Room Document (CRD; 1). The CRD highlighted several key points: there are no analytical methods to determine sweet taste of carbohydrate sources relative to lactose for regulatory compliance of FUF; there are several official methods/standards for analyzing individual carbohydrates or sugar profile in foods, but these methods determine carbohydrate composition and not sweet taste; and sweet taste can be determined by standard sensory analysis methods. However, no sensory intensity reference value for the sweetness of carbohydrate sources can be defined as an indicator for sweetness in FUF because it is unfeasible to define an accurate sweetness reference value or selectively measure perceived sweetness of carbohydrate sources in FUF. In May 2021, CCMAS41 agreed to inform CCNFSDU that there are no known validated methods to measure sweetness of carbohydrate sources and therefore no way to determine compliance for such a provision (2).

The concept of sweetness and its measurement remains an outstanding discussion for the draft CXS for FUF (CXS 156–1987) at CCNFSDU42 in November 2021. This committee must reflect on the conclusions from CCMAS41 and reconsider the provisions that cannot be determined by any known validated methods of measurement. The AOAC Ad Hoc Expert Panel on Sweetness appreciates that a Codex endorsement of a sweetness concept for this revised standard for FUF (CXS 156–1987) could have implications beyond application to this very specific range of products. Thus, the aim of this paper is to present findings and recommendations in relation to the measurement of sweetness, either analytically or by sensory analysis in all types of food products and beverages.

## Chemical Theories of Sweetness Chemoreception

### Sweet-Tasting Molecules and Human Perception

Sweet taste perception in humans is a complex mechanism that starts with recognition of sweet-tasting molecules by G-protein-coupled sweet receptors (T1R2/T1R3) on the surface of taste buds located on the tongue. This recognition initiates biochemical cascades involving the stimulation of protein messengers and subsequent transmission of a neurological signal conveying the intensity and quality of the sweet sensation from taste buds through nerves and finally to the brain (3, 4).

Sweet-tasting molecules (5, 6) can be of natural or synthetic/artificial origin and include carbohydrates, sugar alcohols, and high-intensity sweeteners (HIS). Sweet-tasting carbohydrates are generally simple sugars (mono- and disaccharides) that include glucose, galactose, fructose, maltose, lactose, and sucrose. Some trisaccharides (degree of polymerization, DP = 3) are also known to elicit a sweet taste, including raffinose, maltotriose (7), 4-galactosyl-kojibiose, and lactulosucrose (8). Carbohydrates of DP >3 are generally perceived as starchy rather than sweet. Examples include starches (resistant and digestible), gums, fructans, galacto-oligosaccharides, and dietary fiber. Commercially available maltodextrins often have diverse DP profiles and differ markedly in sweetness despite having equivalent dextrose equivalence (DE) values. In other words, it is possible for two different maltodextrins to have the same DE value but significantly different sweetness. Some examples of sugar alcohols are sorbitol, xylitol, mannitol, lactitol, and maltitol. High-intensity sweeteners include saccharin, acesulfame potassium (Ace-K), sucralose, advantame, and cyclamate; some sweet-tasting terpenoids (e.g., steviosides, glycyrrhizin, and mogrosides); dipeptides (e.g., aspartame and neotame); and some sweet proteins [e.g., brazzein, thaumatin, miraculin, monellin, mabinlin, pentadin, and neoculin (curculin)].

Several models have been developed over the last 50 years to explain the sweetness of molecules but no one model is recognized as the best (9, 10). This is because no one model can accommodate the structural diversity of all sweet-tasting molecules. The original AH-B model suggested that sweet-tasting molecules contain a glucophore moiety that consists of two electronegative atoms at a particular distance from each other. The “AH” acts as a hydrogen bond donor and “B” acts as a hydrogen bond acceptor (11). This model was thought to explain the sweetness of structurally diverse compounds such as β-D-fructose, chloroform, alanine, and saccharine (11). The AH-B motif was later updated to a tripartite glycophore, where a hydrophobic binding site X, was included. In the resultant AH-B-X model, these three groups must exist in a triangular geometry with specific bond lengths to bind with receptors effectively. Hydrogen bonds form at A and B, and X acts as a lipophilic region (12). This model explained why for some amino acids their D-isomers are sweet but L-isomers are not. Examples include D-leucine, D-tryptophan, and D-phenylalanine (12). However, although lipophilicity may enhance sweetness, the inclusion of X was an unnecessary extension as it could not explain the sweetness of glycine and other sugar alcohols (9). The α-helix receptor protein theory was also introduced to explain the structural differences between sweet- and non-sweet-tasting amino acids (13). In addition, the direct G-protein interaction (DGI) theory proposed that non-sugar sweeteners with amphiphilic properties activate G-proteins directly under physiological conditions and this mechanism is consistent with their temporal characteristics such as slow taste onset and lingering aftertaste (14). Finally, the multi-point attachment (MPA) model suggested that a total of eight sites (AH, B, G, D, Y, XH, E1, and E2) interact between a sweet-tasting molecule and the receptor, and although attachment at all eight sites is not required, the resulting number of binding sites involved determines the potency of sweetness (15). This MPA theory was adopted to explain the intense sweetness of neotame and aspartame (16).

### Relative Potency of Sweet-Tasting Molecules

Quantitating sweet taste is not as simple as determining molecular concentrations and extrapolating to a relative scale. Relative sweetness is often reported as sweetness potency, which is calculated as the aqueous concentration of a sweet-tasting molecule to that of another sweet-tasting molecule at equivalent sweetness intensity. Sweetness potency is assessed by human taste panelists and values are most frequently reported relative to sucrose. Using this approach, sucrose is given a value of 1 or 100. A sweetness potency of >1 or >100 is considered as “more potent” than sucrose, since a lower concentration of the sweet-tasting molecule of interest is required to achieve the same sweetness intensity of a specific sucrose concentration. Several methods have been applied to identify equivalent sweetness intensity (17) but the most common are the difference test [e.g., two-alternative forced-choice (2-AFC)], scaling [e.g., Labeled Magnitude Scale (LMS)], and the sucrose-sweetener combined (SSC) method.

The applicability of sweetness potencies reported in the literature is challenged by several practical limitations. Although the relative sweetness values of most sweet-tasting molecules in aqueous solutions are readily available, many of the values are reported as ranges and without noting the temperature of aqueous solution, concentrations of sucrose at which the estimate was made, or which method was applied. Consequently, the determination of sweetness by sensory methods is challenging because it easily leads to ranges of relative sweetness rather than absolute values (Table 1). It is important to understand that the sweetness potency of a sweet-tasting molecule (including sucrose) varies with temperature (Table 2), reference concentration, and evaluation methods (Table 3), and accuracy can be affected by the concentration range evaluated by the panelist (27). Additionally, variation still exists due to the bias of sensory methods and physiological differences among even highly trained panelists. Table 3 lists a few examples of sweetness potency of sweet-tasting molecules relative to different sucrose concentrations in aqueous solution at room temperature, as reported by different authors using different methods.

**Table 1.** Summary of relative sweetness ranges in relation to sucrose equal to 100 (18–21)

Molecule	Relative sweetness
Fructose	80–180
Glucose	50–75
Galactose	54
Lactose	15–40
Maltose	30–50
Trehalose	45
Saccharine	20* *000–70* *000
Acesulfame-K	13* *000–20* *000
Sucralose	40* *000–80* *000
Stevioside	30* *000
Aspartame	12* *000–20* *000
Neotame	700* *000–1300* *000
Thaumatin	200* *000–300* *000
Sorbitol	50–70
Xylitol	90–100
Mannitol	50–70
Lactitol	30–40
Maltitol	80–90
Erythritol	50–80

**Table 2.** Sweetness potency of sucrose at different test temperatures compared to sucrose concentration (5, 10 and 20%) at room temperature^a^^,^^b^

Concentration of sucrose reference at 22°C	5%	10%	20%
5°C (± 2°C)	89	88	92
37°C (± 1°C)	100	100	100
50°C (± 3°C)	108	106	108

a Sweetness of sucrose solutions at room temperature was regarded as 100.

b Data were extracted from Hyvonen et al. (22).

**Table 3.** Sweetness potency of selected sweeteners relative to different sucrose concentrations in aqueous solution, reported by different authors and using different methods (sweetness of sucrose solution was regarded as 1)

Sweetener		Sucrose, % (w/v)		Sweetness potency	Method		Reference
Carbohydrates							
Fructose		5		1.05	2-AFC		(23)
		5; 10; 15		1.25; 1.36; 1.34	LMS		(24)
Dextrose		5; 10; 15		0.64; 0.64; 0.69	LMS		(24)
Xylose		5		0.63; 0.61	2-AFC		(23, 25)
Tagatose		5		0.85	2-AFC		(23)
		3; 5; 10; 15		0.89; 0.89; 0.90; 0.90	LMS		(26)
Allulose		5; 10; 15		0.71; 0.75; 0.80	LMS		(24)
High-intensity sweeteners							
Sucralose		5		500; 561.8	2-AFC	(23, 25)	
		2; 8; 9; 16		740; 414; 430; 194	SSC	(27)	
		3; 5; 10; 15		1896; 954; 376; 218	LMS	(26)	
		5; 10; 15		521; 285; 201	LMS	(24)	
Aspartame		2		182	Ranking method	(28)	
		5; 10		111; 119	2-AFC	(23, 29)	
		5; 10; 15		173; 121; 112	LMS	(24)	
Stevia		5		64	2-AFC	(23)	
		5; 10; 15		348; 263; 181	LMS	(24)	
Reb A		5		144.93	2-AFC	(25)	
		2; 8; 9; 16		263; 46; 46; 27	SSC	(27)	
		3; 5		439; 300	LMS	(26)	
Luo han guo extract		5		75.76	2-AFC	(25)	
		5; 10; 15		262; 144; 106	LMS	(24)	
Acesulfame-K		5; 10; 15		171; 120; 88.1	LMS	(24)	
Sugar alcohols							
Sorbitol		9.12		0.51	Rating method	(30)	
		5; 10; 15		0.80; 0.72; 0.83	LMS	(24)	
Xylitol		5		0.83; 0.98	2-AFC	(23, 25)	
		10; 15		1.01; 1.12	LMS	(24)	
Erythritol		5		0.53	2-AFC	(23)	
		3; 5; 10; 15		0.50; 0.57; 0.70; 0.78	LMS	(26)	
		5; 10; 15		0.72; 0.75; 0.84	LMS	(24)	
Maltitol		5		0.67	2-AFC	(23)	
		5; 10; 15		0.93; 0.89; 0.95	LMS	(24)	
Mannitol	9.12		0.72		Rating method	(30)	
	5; 10; 15		0.58; 0.68; 0.81		LMS	(24)	

Less information is available about sweetness potency of sweet-tasting molecules in food and beverage matrixes (as compared to aqueous solutions). This is because sweetness is not absolute but rather depends on concentration, pH, serving temperature, matrix effects (e.g., presence of other molecules that affect mineral content and viscosity), and synergistic effects (i.e., presence of other sweet-tasting molecules) (29, 31, 32). In addition, the perception of sweetness intensity varies with time for certain foods and beverages (e.g., lingering sweetness or bitterness of most HIS). As a result, it is unfeasible to determine a sweetness potency value of a sweet-tasting molecule by comparing it to aqueous sucrose solution and applying the value to more complex food and beverage matrixes. Instead, different sensory methods with highly trained panelists are needed to assess the sweetness of sweet-tasting molecules in each unique food and beverage matrix.

## Analytical Methods for Quantitating Sweet-Tasting Molecules

To the best of our knowledge, there are no stand-alone analytical methods for determining the sweetness of sweet-tasting molecules in food products and beverages. On the other hand, an abundance of analytical methods for quantitating the composition of these molecules in food products and beverages has been reported in the literature. The most relevant and current analytical techniques and official methods are discussed in this section, with an emphasis on methods for quantitating carbohydrates and relevant sweet-tasting molecules in ingredients and final products.

**Figure 1.**
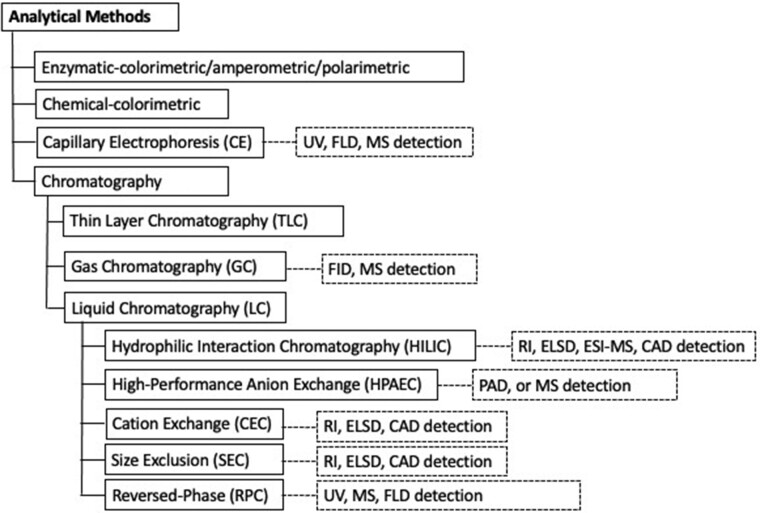
Overview of the most common analytical methods for quantitating natural and artificial sweet-tasting molecules.

**Figure 2.**
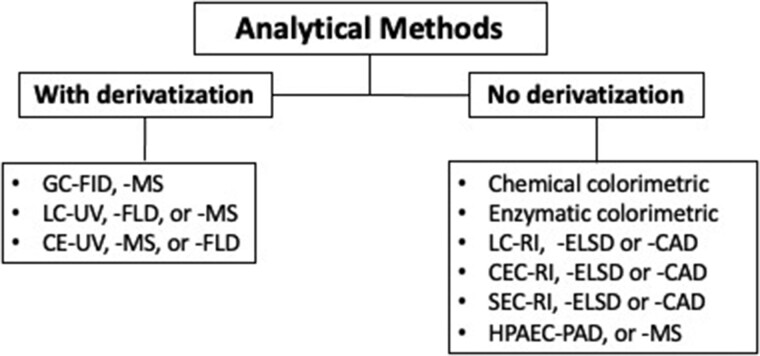
Examples of analytical methods for which derivatization/labeling is or is not needed.

A variety of analytical techniques are available for both fundamental analytical research and standard routine quantitation (Figure 1). Since most carbohydrates lack a strong UV chromophore, fluorophore, or charge, derivatization/labeling with a suitable molecule may be necessary, depending on the separation and detection techniques applied (Figure 2) (33–36). Most state-of-the-art methods use well-accepted analytical instruments to selectively determine a single carbohydrate or multiple carbohydrates (i.e., sugar profile methods that generally include two or more of the most common mono- and disaccharides—glucose, fructose, galactose, lactose, sucrose, and maltose) in ingredients and finished (food) products. Specifically, chromatographic methods like high-performance anion-exchange chromatography with pulsed amperometric (HPAEC–PAD; 37, 38) or mass spectrometric detection (HPAEC–MS; 39), and HPLC with tandem mass spectrometric (HPLC–MS/MS; 40), evaporative light-scattering (HPLC–ELS; 41), refractive index (HPLC–RI; 42), or charged aerosol detection (HPLC–CAD; 43, 44) have been used. These techniques continue to be developed and optimized to extend their applicability to various complex food matrixes while providing good accuracy and precision, high sensitivity and resolution, and required LOD and LOQ. In parallel, classical (chemical) methods based on colorimetry (e.g., phenol-sulfuric acid, Lane-Eynon, Luff Schoorl, Somogyi-Nelson, etc.) or calculation (e.g., Method **986.25**) of total carbohydrates and sugars are still applied. Alternatively, highly specific enzyme-based methods (45), including enzymatic–amperometric (e.g., Method **2020.01**) and enzymatic–polarimetric (e.g., Clerget method, IS 11764:2005/ISO 2911:2004) techniques are also well established and validated for the measurement of individual sugars or groups of sugars. Furthermore, NMR (46) and capillary electrophoresis (CE) have also been suggested as useful tools for carbohydrate determination in food products (47) or beverages (48).

For both natural and artificial sweeteners, a variety of official standard methods are available based on different analytical chemical techniques. Tables 4–9 summarize the most important and currently applied methods. Of these methods, enzymatic–colorimetric and liquid chromatographic techniques are most often applied to the standard analysis of carbohydrates.

**Table 4.** AOAC *Official Methods*^SM^ for quantitative analysis of natural and artificial sweeteners

Method	Commodity	Provision	Principle	Codex type
**923.09**	Sugars and syrups	Invert sugar	Volumetric (Lane-Eynon)	—^a^
**970.58**	Molasses	Invert sugar	Titrimetry	—
**971.17**	Cyclamates and artificially sweetened products	Cyclohexylamine	Infrared spectroscopy	—
**973.29**	Foods	Saccharin	Gravimetric	—
**978.17**	Honey	Corn and cane sugar products	Carbon isotope ratio MS	I^b^
**984.15**	Milk	Lactose	Enzymatic	—
**985.09**	Wine	Glucose, fructose	Enzymatic	—
**986.25**	Infant formula	Total carbohydrates	Calculation	I
**988.12**	Raw cane sugar	Dextran	Colorimetry (Roberts Copper)	—
**992.09**	Fruit juices/frozen concentrated orange juice	Syrups/sugar beet-derived syrup	MS	—
**995.13**	Instant coffee	Carbohydrates	HPAEC–PAD	—
**995.17**	Fruit juices	Beet sugar	NMR spectroscopy	—
**996.04**	Cane and beet final molasses	Sugars (glucose, fructose, sucrose)	LC	—
**998.12**	Honey	Sugars added (C-4 sugars)	Carbon isotope ratio MS	I^c^
**2000.17**	Raw cane sugar	Trace glucose and fructose	Anion-exchange chromatography	—
**2000.19**	Maple syrup	Beet or cane sugar	NMR spectroscopy	—
**2006.06**	Milk	Lactose	Spectrophotometric–enzymatic	—
**2013.12**	Wine and wine-like products	Total carbohydrates	HPLC–RI	—
**2018.16**	Food, dietary supplements, pet food, and animal feeds	Sugar profile	HPAEC–PAD	—
**2020.01**	Dairy products/milk	Lactose	LactoSens^®^ Amperometric	—
**2020.07**	Cereals and cereal products, dairy products, vegetables, fruits, and fruit products	Available carbohydrates	Enzymatic	—
**2020.08**	Low-lactose, lactose-free dairy products, and conventional dairy products	Lactose	Enzymatic	—

a — = Not applicable.

b CXS 234–1999 refers to Method **978.17** which has been replaced by Method **998.12**.

c CXS 234–1999 refers to Method **998.18**. The authors were not able to identify the Method **998.18** and believe the correct method to be Method **998.12**.

**Table 5.** Official ISO and ISO | IDF methods for quantitative analysis of natural and artificial sweeteners

Method	Commodity	Provision	Principle	Codex type
ISO 2911 | IDF35	Sweetened condensed milk	Sucrose	Polarimetry	IV
ISO 5377	Starch and hydrolysis products	Reducing power and dextrose equivalent	Titrimetry (Lane-Eynon)	I
ISO 5548 | IDF 106	Edible casein products	Lactose	Photometry (phenol and H_2_SO_4_)	IV
ISO 5765–1/2 | IDF 79–1/2	Whey powders	Lactose	Enzymatic: Part 1—Glucose moiety or Part 2—Galactose moiety	II
ISO 10504	Glucose, fructose-containing and hydrogenated glucose syrups	Glucose, maltose, maltotriose, fructose, sorbitol, mannitol, maltitol, and malto-oligosaccharides	HPLC–RI	II^a^
ISO 11285 | IDF 175	Milk	Lactulose	Enzymatic	—^b^
ISO 11292	Instant coffee	Free and total carbohydrates	HPAEC–PAD	—
ISO 11868 | IDF 147	Heat-treated milk	Lactulose	HPLC	—
ISO 22184 | IDF 244	Milk and milk products	Galactose, glucose, fructose, sucrose, lactose, and maltose	HPAEC–PAD	—
EN ISO 22579 | IDF 241	Infant formula/adult nutritionals	Fructans	HPAEC–PAD	—
ISO 22662 | IDF 198	Milk and milk products	Lactose	HPAEC–PAD	—
ISO 26462 | IDF214	Milk	Lactose	Enzymatic	—

a CXS 234–1999 refers to ISO 10504 as Type II method for the commodity sugars (fructose) with either glucose or fructose as provisions.

b — = Not applicable.

**Table 6.** Official EN/CEN, CEN/TS (Technical Specifications) and NMKL methods for quantitative analysis of natural and artificial sweeteners

Method	Commodity	Provision	Principle	Codex type
EN 1140/IFUMA 55	Fruit juices and nectars	Glucose and fructose	Enzymatic	II
EN 12146/IFUMA 56	Fruit juices and nectars	Sucrose	Enzymatic	III
EN 12630/IFUMA 67/NMKL 148	Fruit juices and nectars	Glucose and fructose	HPLC	III
EN 12630/IFUMA 67/NMKL 148	Fruit juices and nectars	Sucrose	HPLC	II
NMKL 122	Fruit juices and nectars	Saccharin	LC	II
NMKL 123	All foods	Cyclamate	Spectrophotometry	III
EN 12856	All foods	Acesulfame-K, aspartame	HPLC	II
EN 12856	All foods	Saccharin	HPLC	III
EN 12857	All foods	Cyclamate	HPLC	II
EN 1376	Table-top sweeteners	Saccharin	Spectrometric	III
EN 1377	Table-top sweeteners	Acesulfame-K	Spectrometric	II
EN 1378	Table-top sweeteners	Aspartame	HPLC	II
EN 1379	Liquid table-top sweeteners	Cyclamate and saccharin	HPLC	II
CEN/TS 14537	Foodstuffs	Neohesperidin-dihydrochalcone	HPLC	—^a^
CEN 15086	Foodstuffs	Isomalt, lactitol, maltitol, mannitol, sorbitol, and xylitol	HPLC	—
CEN 15606	Foodstuffs	Acesulfame-K, aspartame, Neohesperidin-dihydrochalcone, and saccharin	HPLC	—
CEN/TS 15754	Animal feeding stuffs	Sugars	HPAEC–PAD	—
CEN 15911	Foodstuffs	Sweeteners	HPLC–ELSD	—

a — = Not applicable.

**Table 7.** Official AACC International and International Association for Cereal Science and Technology (ICC) methods for quantitative analysis of natural and artificial sweeteners

Method	Commodity	Provision	Principle
ICC 132	Cereals and cereal products	Saccharose	Enzymatic
AACC Method 80–04.01	Cereals	Fructose, glucose, sucrose, maltose, and lactose	HPLC
AACC Method 80–05.01	Corn syrups, fructose-containing syrups, corn sugars, and starch hydrolysates	Saccharides	LC
AACC Method 80–10.01	Sugar mixture	Glucose	Enzymatic
AACC Method 80–50.01	Feeds and feedstuffs	Sucrose	
AACC Method 80–60.01	Flour and semolina	Reducing and nonreducing sugars	
AACC Method 80–68.01	Prepared bakery mixes	Reducing sugars	Titrimetry (Luff Schoorl)

**Table 8.** Official ICUMSA methods for quantitative analysis of natural and artificial sweeteners

Method	Commodity	Provision	Principle	Codex type
GS1-3	Cane raw sugar	Reducing sugars	Titrimetry (Lane-Eynon)	—^a^
GS1/3/7–3	Sugars (soft white and soft brown sugar)	Invert sugar	Titrimetry (Lane-Eynon)	I
GS1/3/7–3	Sugars (plantation or mill white sugar)	Invert sugar	Titrimetry (Lane-Eynon)	I
GS1-4	Raw sugar	Glucose, fructose	HPAEC	—
GS1-5	Cane raw sugar	Reducing sugars	Titrimetry (Luff Schoorl)	—
GS2-4	White sugar	Glucose, fructose	Enzymatic (hexokinase method)	—
GS2-5	White sugar	Reducing sugars	Titrimetry (Knight-Allen EDTA method)	—
GS2/3–5	Sugars (powdered sugar)	Invert sugar	Titrimetry	I
GS2-6	White sugar	Reducing sugars	Titrimetry (modified Ofner method)	—
GS4-1	Molasses	Apparent sucrose	Double polarization method	—
GS4-2	Molasses, factory products, and cane juice	Sucrose	GC	—
GS4-3	Cane molasses	Reducing sugars	Titrimetry (Lane-Eynon)	—
GS4/3–3	Sugars (lactose)	Anhydrous lactose	Titrimetry	II
GS4/3–3^b^	Sugars (soft white and soft brown sugar)	Invert sugar	Titrimetry (Lane-Eynon)	I
GS4-5	Beet molasses	Reducing sugars	Titrimetry (Lane-Eynon)	—
GS4-7	Molasses and refined syrups after hydrolysis	Total reducing sugars	Titrimetry (Lane-Eynon)	—
GS4/3–7	Sugars (soft white and soft brown sugar)	Sucrose plus invert sugar	Titrimetry	I
GS4-9	Molasses and refined syrups after hydrolysis	Total reducing sugars	Titrimetry (Luff-Schoorl)	—
GS4-22	Beet molasses	Sucrose and betaine	HPLC	—
GS7-22	Cane juices, syrups, and molasses	Fructose, glucose, sucrose	GC	—
GS7-23	Cane molasses	Fructose, glucose, sucrose	HPLC	—
GS7-23	Beet molasses	Sucrose	HPLC	—
GS7-24	Cane juices, syrups, and molasses	Glucose, fructose, sucrose	High performance ion chromatography (HPIC)	—
GS7-24	Beet molasses	Sucrose	HPIC	—
GS8-4	Beet juices and processing products	Glucose, fructose	Enzymatic	—
GS8-5	Beet pulp	Apparent total sugar content	Titrimetry (Luff-Shoorl)	—
GS4-18	Beet molasses	Total α-galactosides and raffinose	Enzymatic	—
GS14-19	Beet molasses	Raffinose	HPAEC	—

a — = Not applicable.

b Applicable at levels >10% w/w.

**Table 9.** Official Chinese GB, and China CIQ Import Commodity Inspection Standards(SN) methods for quantitative analysis of natural and artificial sweeteners

Method^a^	Commodity	Provision	Principle
GB/T 5009.7–2008	Foods	Reducing sugars	Titrimetry
GB/T 5009.7–2016	Foodstuffs	Reducing sugars	Titrimetry
GB 5009.8–2016	Foods	Fructose, glucose, sucrose, maltose, and lactose	HPLC
GB 5413.5–2010	Foods for infants, young children, milk, and milk products	Lactose, sucrose	HPLC
GB/T 5513–2008	Foods	Reducing sugars	Titrimetry
GB/T 5513–2019	Grains	Reducing sugars	Titrimetry
GB/T 9695.31–2008	Meat products	Total sugars	Spectrophotometry and titrimetry
GB/T 37493–2019	Cereals and pulse seeds	Soluble sugars	Titrimetry (Shaffer-Somogyi)
SN/T 3538–2013	Foodstuffs for export	Cyclamate, sodium saccharin, acesulfame, aspartame, alitame, and neotame	LC–MS/MS
SN/T 3850.1–2014	Foods for export	Sugar alcohol sweeteners	LC–MS and ion chromatography
SN/T 3850.2–2014	Foods for export	Sugar alcohol sweeteners	GC

a Codes without T are mandatory, codes with T are recommended.

Three basic strategies are used for the enzymatic analysis of monosaccharides: Different LC systems are often applied in standard analysis protocols for sugars, carbohydrates, carbohydrate derivatives, and artificial sweeteners. In the past, cation-exchange LC in combination with RI detection was applied for the measurement of mono- and disaccharides in relatively simple food matrixes (e.g., Method **980.13**, ISO 10504, ISO 11868/IDF 147, ISO 22662 | IDF 198). However, presently, sugar profiles in human foods and animal feeds are measured with HPAEC–PAD [e.g., Method **2018.16**, Method **995.13**, ISO 22184 | IDF 244, The European Committee for Standardization (CEN) 15754)] because of its greatly improved resolution and sensitivity. Additionally, HPAEC–PAD does not require precolumn derivatization/labeling, making it a strong tool in carbohydrate quantitation (e.g., Method **995.13**, Method **2018.16**, ISO 11292, ISO 22184 | IDF244, and ISO 22579 | IDF 241). Chromatographic techniques are mainly applied for the quantification of artificial sweeteners in food products [e.g., European Standards (EN)] 1378, EN 1379, EN 12856, EN 12857, and CEN 15606), although official gravimetric methods such as Method **957.10** and Method **973.29** for cyclamate and saccharin determination are also available.

1. phosphorylation of the sugars followed by oxidation of glucose 6-phosphate and concurrent reduction of nicotinamide adenine dinucleotide (NAD^+^) or nicotinamide adenine dinucleotide phosphate (NADP^+^) to NADH or NADPH, which are measured colorimetrically;
2. direct oxidation of sugars (e.g., galactose or xylose) by dehydrogenase and concurrent reduction of NAD^+^ or NADP^+^ to NADP or NADPH, which has also been applied to a range of sugar alcohols such as sorbitol/xylitol and mannitol/arabitol; and
3. oxidation of sugars and concurrent production of hydrogen peroxide, which can be linked to a colorimetric detection system. Disaccharides are usually measured after hydrolysis to constituent monosaccharides with dedicated enzymes (e.g., Method **2020.08**, Method **2020.07**, Method **2006.06**, ISO 26462 | IDF 214).

Generally, methods have been validated and applied specifically for a single or limited number of ingredients and/or food products. For example, methods have been validated for products such as milk/milk products and infant formula (ISO 22184 | IDF 244), foods of low/high protein or sugar matrixes (Method **2018.16**), fruit/fruit juices (Method **971.18**), cereals (Method **982.14**), milk chocolate (Method **980.13**), and instant coffee (Method **995.13**, ISO 11292). In addition to the aforementioned sugar profile methods, there are official methods for quantitating lactose in raw/processed milk (Method **2006.06**) and lactose-free or low-lactose dairy products and milk (Method **2020.01**), and lactose and sucrose in foods for infants and young children and milk and milk products [GuoBiao Standards (GB)] 5413.5–2010). Furthermore, methods for determining complex carbohydrates (i.e., those that are not particularly sweet) in relevant commodities are also available. These include, among others, fructans in foods, pediatric nutritional formula, and infant formula (Method **997.08**, Method **999.03**, Method **2016.14**, Method **2016.06**, ISO 22579 | IDF 241); galactooligosaccharides in foods, cereals, dairy products, and infant formulas (Method **2001.02**, Method **2021.01**); and β-glucans in barley and oats [Method **995.16**/Cereals & Grains Association (AACC) 32–23-01/Codex Type II)].

CXS 234–1999 lists several Type I, II, and III methods for quantitation of natural and artificial sweet-tasting compounds in various commodities. Among these are Type II methods for glucose and fructose determination in fruit juices and nectars [EN 1140/International Fruit and Vegetable Juice Association Methods of Analysis (IFUMA) 55] and sugar (fructose) commodities (ISO 10504:1988); lactose in sugar (lactose) commodities [International Commission for Uniform Methods of Sugar Analysis (ICUMSA) GS 4/3–3 (1994)] and whey powders (ISO 5765–1/2 | IDF 79 1/2); acesulfame-K and aspartame in all foods (EN 12856) as well as table-top sweeteners (EN 1377 and EN 1378 for acesulfame-K and aspartame, respectively); cyclamate in all foods (EN 12857) and liquid table-top sweetener preparations (EN1379); and saccharin in fruit juices/nectars [Nordic Committee on Food Analysis (NMKL) 122] as well as liquid table-top sweetener preparations (EN1379). Additionally, CXS 234–1999 includes Type III methods for glucose and fructose in fruit juice and nectars (EN 12630/IFUMA 67/NMKL 148); carbohydrates in food for special dietary uses [method described in Codex Alimentarius Commission (CAC)/VOL IX-Ed.1, Part III)]; cyclamate in all foods (NMKL 123); and saccharin in all foods (EN 12856) and table-top sweeteners (EN 1376).

To our knowledge, there are no official methods for quantitating sweet glycosides such as steviosides and mogrosides in food products. However, various methods for the determination of these sweet-tasting molecules in leaves and fruit extracts and table-top sweeteners have been published. Examples of techniques include enzymatic approaches (49); LC (50–53); near infrared spectroscopy (NIR; 54–56); LC–MS/MS (57, 58); HPTLC (59–61); and hydrophilic interaction LC (HILIC) with charged aerosol and UV detection (62, 63). The Joint FAO/WHO Expert Committee on Food Additives (JECFA) recommends an HPLC-based method for the estimation of steviol glycosides in stevia (64). Furthermore, Fayaz et al. (65) described the determination of stevioside and rebaudioside-A in milk, yogurt, chewing gum, jam, and carbonated water by HPLC–UV, while Wald et al. (66) published an HPTLC method for determination of steviosides in food products, stevia leaves, and formulations.

## Sensory Evaluation of Sweetness in Foods and Beverages

### Sensory Evaluation Principles

Sensory evaluation methods that include discrimination, descriptive, and affective tests have been developed to measure and analyze human responses to foods and beverages as perceived through the sense of sight, smell, touch, taste, and hearing (67, 68). The choice of test methods depends on the purpose of analysis. For example, discrimination tests are used to determine if products are different, while descriptive tests focus on the degree to which products are different with respect to specific sensory characteristics. Affective tests are usually applied when the objective is to determine how well products are liked or which products are preferred. The required number of panelists can vary from 8 to 200 depending on the method. For a scenario in which quantitative descriptive tests are used (69), panelists usually receive extensive training, varying between 10 and 120 hours depending upon the complexity of the product aroma, flavor, and texture profile. Training aims to build a sensory glossary (70) and calibrate panelists on perceived intensity of each attribute, which are quantified on an intensity scale by a panel of experts. Once selected, they are trained to perceive, identify, and rate the perceived intensity of each attribute elicited by the product of interest (71). Extensively trained panelists are qualified as experts (71).

To ensure quality results, sensory evaluation is performed in sensory booths to prevent participants from interacting and influencing each other’s judgments. A randomized and balanced order of product presentation helps statistically suppress carryover effects (i.e., influence of one stimulus on perception of subsequently evaluated stimuli; 69). Finally, the number of stimuli evaluated in a single session is defined per product category to avoid sensory fatigue and saturation.

### Sensory Methods for Measuring Sweetness

There are several commonly applied methods for measuring food or beverage perception using trained human panels (72, 73), all of which are based on the identification and quantitation of perceived sensations by adequately trained assessors. Sensory descriptive analysis methods all rely on the consideration that even though perception is subjective and varies from one individual to another, intensive training of panelists reduces this inter-individual variability. A common frame of reference is shared and learned by all panelists during training, so they are able to score the sensations they perceive in a similar way. These references are purposely chosen to illustrate, in a qualitative and quantitative manner, the levels of sweetness intensity that can be encountered in the product category under consideration. However, none of these methods are specifically designed for measuring only sweetness in food. Instead, they are designed to cover all sensory characteristics perceived when eating or drinking a product. These characteristics range from appearance to smell, texture, and taste properties. This also includes basic tastes like sweetness as well as flavors perceived by retro-nasal olfaction.

Among available methods, several can be considered the most relevant for measuring sweetness in FUF and other food products and beverages. These include magnitude estimation as described in ISO 11056; studies using a specific measurement scale; the LMS; and quantitative descriptive sensory profile as described in ISO 13299 [including Quantitative Descriptive Analysis^®^ (QDA^®^) and Spectrum™ methods]. These methods have been developed to identify and quantify the sensory characteristics of a product in its entirety (i.e., all the properties of a product perceived using the five senses). Therefore, according to the objective of the study, the panel would either be restricted to measure the perceived sweetness of different food products or beverages, or would include sweetness and other sensory characteristics that would presumably be interacting with sweetness. Moreover, these methods are designed to discriminate between different products within the same category. A comparison pair might be a newly developed product versus a competitor product, two existing recipes, or for comparison to a fixed reference. This reference would consist of a product with known composition to allow reproduction and validation across tests/comparisons. The reference product would represent the maximum threshold of sweetness that every newly developed product should not exceed.

Although the methods mentioned above are all relevant for measuring perceived sweetness of FUF and other food products and beverages, each requires different investments of time and resources. Therefore, a sensory scientist should consider several criteria to select the most appropriate method for their objectives, including number of panelists to be recruited; screening process requirements; sensory properties to be evaluated; time duration of training; panel performance validation; and duration of panel availability (e.g., how many studies and for how many months).

### Magnitude Estimation Method

This method can be considered the “gold standard” for ratio-level measurement of intensity and has historically been the first method used for measuring relative sweetness of different carbohydrate sources in aqueous solutions. The notion of “ratio” refers to the proportionality that two samples may display on a specific sensory property. For example, a carbohydrate source with a sweetness intensity of four can be considered twice as sweet as another carbohydrate source with a sweetness intensity of two.

### Labeled Magnitude Scale

The LMS uses a non-linear, continuous scale graduated in a quasi-logarithmic way, with each graduation translating a level of perceived intensity. Scores of perceived sweetness could thus be indicated on this scale from “no sweetness at all” to “the strongest sweetness imaginable.” Empirical data are based on ratio-scaling and similar to those from magnitude estimation.

### Quantitative Descriptive Sensory Profile

In the quantitative descriptive sensory profile, assessors evaluate samples on a common list of attributes and score their intensity. There are several methods for establishing a quantitative descriptive sensory profile, among which some techniques have been trademarked. Results shall consist of intensity scores for each attribute that can be submitted to univariate analyses. Empirically derived profiles are panel and product category specific and cannot be interpreted by other groups if no reference standards are given.

### Quantitative Descriptive Analysis

This approach is a variant of the quantitative descriptive sensory profile and can be used for a wide variety of purposes, including understanding product similarities and differences, ingredient substitution, new product development, competitive assessments, claims substantiation, advertising, etc. Quantitative Descriptive Analysis uses an unstructured or semi-structured (6 in./approximately15 cm) line scale, anchored 0.5 in. from either end for measuring and scaling perceived differences and intensities. These equal-interval scales are described in psychophysics literature.

### Spectrum Method

The Spectrum method is based on a descriptive profiling procedure that includes using documented references for both qualitative attributes and intensity scale points. The method has precise steps and procedures at every stage of development. This includes selection of assessors to panel leadership, panel training, validation, and maintenance of the panel after training is complete. These practices lead to a descriptive panel that produces reproducible and statistically robust data across multiple sessions and categories. Sensory attributes are identified with both physical external references and written definitions, which should allow describing and discriminating among samples in the product category.

The Spectrum method scale is based on a 0- to 15-point intensity scale with the ability to rate in increments of tenths for 150 points of discrimination. This gives the assessors the ability to discriminate using smaller points of difference. The Spectrum scale is universal, covering the entirety of intensities within a scope as large as the global food system.

### Human Variability in Sensory Response to Sweet Taste

Even with a well-designed study and use of trained panelists, variability among individual panelists exists in sensory response for perceived sweetness (i.e., in perceived intensity rating) due to inherent physiological and psychological differences. Sweet taste threshold differs between individuals due to genetic differences (74, 75) that are modulated by physiological factors such as hormonal mechanisms and taste bud abundance. See Trius-Soler et al. for a systematic review and meta-analysis on this topic (76). Psychological factors such as mood (77) and emotions (78) can also impact sweet taste perception.

Because of the role of physiological and psychological factors in taste perception variation, sweetness intensity is generally represented by an average value and a within-panel variability statistical estimator such as a confidence interval. Therefore, perceived intensity for a given attribute is not a fixed number but rather a number plus or minus the variability statistical estimator value.

### Contextual Factors Contributing to Sweetness Perception

In addition to human variability, contextual factors can also influence perception of sweet taste and increase variability of measured sweetness. Particularly, other taste stimuli generated by food or beverage ingredients such as sourness of organic acids or bitterness of peptides can modulate sweetness perception through binary taste–taste interaction (79). Also, food or beverage sensory modalities such as appearance, smell, and texture (intrinsic contextual factors) or attributes of serving vessels such as cup or plate color, texture, or shape (extrinsic contextual factors) may modulate perceived sweetness through cross-modal perceptual interactions. See Wang et al. for a review (80).

The origin of perceptual interaction is cognitive and built through repeated exposure to sensory stimuli collectively present in beverages or foods. One example is the strawberry aroma and sweet taste experienced during strawberry fruit consumption as well as any sweet foods and beverages flavored with strawberry (81, 82). This so- called associative learning is integrated at a neural level in the orbitofrontal cortex, a brain region responsible for stimulus–stimulus association and integration of sensory perception that helps explain how a strawberry or vanilla odor can enhance perceived sweetness (83, 84).

The magnitude of contextual effects on perception of sweetness differs between people according to their previous food experience. This is true even for a trained panel. As such, associative learning is a resistant phenomenon (85).

### Measuring Sweetness Elicited by an Ingredient in a Finished Food Product or Beverage

Sensory methods are capable of qualifying and quantifying the perceived sweetness of individual ingredients and finished food products and beverages. However, it is unfeasible to selectively measure the perceived sweetness of an ingredient (e.g., carbohydrate source) in a finished product. Even with a well-designed study and training to limit variability between panelists, it is still impossible to define a standard reference value of perceived sweetness intensity as a QC indicator for a specific ingredient in a finished product; especially one that is identical over time and across global taste panels. This is true for several reasons. First, inherent variability in human sensory response contributes variability in perceived intensity rating. Second, the perceived sweetness of an ingredient dissolved in aqueous solution at a given concentration does not necessarily indicate equivalent sweetness for the same ingredient at the same concentration in a finished product because contextual factors like manufacturing processes and other ingredients may modulate sweetness. Consequently, for Codex, selecting “carbohydrate sources that have no contribution to [food or beverage] sweet taste” and “in no case be sweeter than lactose” (CXS 156–1987, Section B. 3.1, footnote 5) cannot be perceptually demonstrated.

### Advancements in Sensory Evaluation

Novel digital technologies have been implemented in sensory science, combining electronic sensors (e.g., e-tongue and e-nose) and artificial intelligence to predict food sensory properties (86). Commercially available e-tongue devices rely on selective receptors to measure sweetness of known compounds like sucrose. Examples include the Alpha MOS, ASTREE Electronic Tongue (Toulouse, France) and Valiber Swizzle 1.0 (Tel Aviv-Yafo, Israel). However, these devices would not be appropriate for verifying compliance with a sweetness regulation due to the inability to quantify the modulation of sweet taste perception induced by other ingredients in a finished product. Also, they are incapable of achieving the accuracy and precision required of an internationally validated standard method. Hence, to our knowledge, no technical solution exists to measure perceived sweetness solely generated by a source of carbohydrate in food products or beverages.

## Conclusions and Recommendations

The Codex Alimentarius Procedural Manual includes procedures for the elaboration of Codex standards and related texts as well as presents the format for Codex commodity standards. One of the required chapters in a Codex standard is “Methods of Analysis and Sampling,” which should contain the following language: “For checking the compliance with this standard, the methods of analysis and sampling contained in the Recommended Methods of Analysis and Sampling (CXS 234–1999) relevant to the provisions in this standard, shall be used.” This language is included in the current FUF standard (CXS 156/1987).

In 2013, the Codex Alimentarius adopted a guideline describing principles for the use of sampling and testing in international food trade (CAC/GL 83–2013). These principles are intended to assist governments in the establishment and use of sampling and testing procedures for determination on a scientific basis, whether foods in international trade comply with specifications. To select appropriate sampling and testing procedures, the guideline states that methods should be fit for the intended purposes and applied consistently.

CCNFSDU has proposed requirements for sweetness of FUF in a revised version of the Codex FUF standard (CXS 156/1987). Consequently, questions have been raised about the ability to measure and enforce a requirement for sweet taste objectively.

The results of a thorough review indicate that there are no analytical methods available for objectively determining the sweetness of sweet-tasting molecules in food products and beverages. An abundance of analytical methods are available, however, to quantitate the composition of these molecules in food products and beverages. In the specific area of sensory evaluation, sweet taste can be determined by standard sensory analysis methods. However, it is impossible to define an accurate reference value for sweetness intensity, which makes it impossible to assess sweetness accurately across global taste panels. Furthermore, it is impossible to selectively measure perceived sweetness of carbohydrate sources in food products and beverages, including FUF, due to taste perception of other ingredients in a finished product matrix. CCMAS confirmed that there are no known validated methods to measure sweetness of carbohydrate sources during its 41st session in May 2021.

Novel digital technologies combining electronic sensors and artificial intelligence are in development. However, these will be unable to measure a perceived sweetness solely generated by an ingredient in food products and beverages. Additionally, these technologies are incapable of achieving the accuracy and precision required of an internationally recognized standard method, especially one that would be considered for adoption in CXS 234–1999.

Considering the need for fit-for-purpose testing procedures to enable verification of compliance with specifications to support international trade, it is not recommended to include any requirement related to “sweetness” or “sweet taste” in a revised Codex standard for FUF or any other food commodities in the future. If, however, there is a need to establish additional requirements beyond those already drafted, considerations should be given on the availability of analytical methods for regulatory compliance verification.
